# Pattern and Outcome of Colorectal and Perianal Surgery in a Referral Hospital, Addis Ababa, Ethiopia

**DOI:** 10.4314/ejhs.v31i5.10

**Published:** 2021-09

**Authors:** Daniel Zemenfes Ashebir, Hana Abebe Gebreselassie

**Affiliations:** 1 College of Health Sciences, Addis Ababa Univeristy, Department of Surgery; 2 St. Paul's Hospital Millenium Medical College, Department of Surgery

**Keywords:** Colorectal surgery, perianal surgery, morbidity, mortality, relaparotomy

## Abstract

**Background:**

Colorectal and perianal surgery encompasses a broad range of procedures to address various pathologies arising from the colon and anorectum. Data regarding the pattern and outcome of colorectal and perianal disorders requiring surgery remains largely unknown in Ethiopia.

**Methods:**

A descriptive cross sectional one-year review was made and all patients who were admitted and operated for colorectal and perianal disorders were included in the study. Data was collected by chart review and analyzed by SPSS version 23.

**Result:**

Colorectal and perianal surgeries accounted for 14.6% of the procedures in the study period. Males constituted the major share of the study population. The mean age for perianal and colorectal pathologies were 39.6±13.7 and 44.8± 16.2 years respectively. Among the colorectal disorders redundant sigmoid was the most common indication for admission 41(34.4%) followed by colorectal neoplasms 38(31.9%) while among the perianal conditions, fistula in ano was the most common pathology accounting for 69(43.4%) of admissions followed by hemorrhoids 35(22%). The overall incidence of post-operative complications in the colorectal and perianal procedure groups was found to be 29(24.4%) and 4(2.5%) respectively. There was no mortality in the perianal group whereas there were 11(9.2%) deaths in the colorectal procedure group.

**Conclusion:**

Colorectal surgeries accounted for a fair share of procedures among the other specialty units. The morbidity and mortality associated with colorectal procedures is fairly high and warrants attention.

## Introduction

Colorectal and perianal surgery encompasses a broad range of procedures to address various pathologies arising from the colon and anorectum including congenital disorders, inflammatory conditions of the colon, diverticular disease, trauma, malignancy and perianal conditions.

Colorectal and perianal surgery has its roots in the early civilization starting from the ancient Egyptians. The first surgical treatments for colorectal and perianal diseases were limited to rectal prolapse and hemorrhoids but later its evolution progressed through time to include other pathologies related to trauma and malignancy. The last century was a time of marked progress with the introduction of minimally invasive approaches namely laparoscopic and robotic procedures to the practice of colorectal and perianal surgery (1).

Historically, colorectal surgery was associated with a high risk of morbidity and mortality compared to other surgeries which is in the range of 13–28%. This high rate of complication has been decreasing over the decades due to betterment in the availability of broad-spectrum antibiotics, development of improved surgical techniques and advances in critical care and perioperative management of patients (2). Common complications following colorectal procedures include bleeding, iatrogenic injury to adjacent organs, wound related complications, anastomotic leak, prolonged ileus and thromboembolism (3).

Several patient factors and perioperative variables predict the occurrence of complications after colorectal surgery. Among these factors are emergency surgery, presence of comorbidities, high ASA (American Society of Anesthesiology) grade, malnutrition, anemia, intraoperative blood transfusion, use of steroids, albumin <3.5 g/L and creatinine >1.4 mmol/L, longer operative time (> 120 minutes), and peritoneal contamination (4–6). The burden and pattern of colorectal disorders in Ethiopia is largely unknown although there are few hospital-based reports. In one study from Tikur Anbessa Specialized Hospital, colorectal pathologies accounted for 7.2% of all elective admissions in the surgical department (7). In another report from North western Ethiopia surgical pathologies of the colon accounted for 5.4% of the admissions and 7% of the annual surgical procedures being the third most common procedure among the other specialty surgeries (8). In addition, in a three years review of admission pattern to General surgery and Urology wards in St. Paul's Hospital Millennium Medical Collage, colorectal and perianal pathologies were responsible for 8.4% of the emergency and 19.6% of the elective admissions (9).

The objective of this study is to describe sociodemographic characteristics, admission diagnosis, the types of procedures and patient outcome of colorectal and perianal surgeries in Menelik II referral hospital. This will provide a vital input to improve the level of surgical care to patients and lay foundation for further research.

## Materials and Methods

This study was conducted in Menelik II Referral Hospital which serves patients from the metropolis and surrounding towns. The surgical department of the hospital consists of one colorectal surgeon, one cardiothoracic surgeon, four general surgeons, one urology surgeon, one neuro surgeon and surgical residents from Addis Ababa University.

Retrospective review of charts of patients operated for colorectal disorders in the time period of January 1, 2018 to December 31, 2018 was done to determine the pattern and outcome of colorectal surgery at Menelik II referral Hospital. As the number of retrieved charts in the one year of the study period was equal to the calculated sample size (240 with 10% added to account for incomplete and lost charts, P=.054 taken from study from Gonder, E=.03 and Z=1.96) all patients admitted and operated to surgical wards in the given period were included in the study. The colorectal surgeries which are included in this paper were performed by the colorectal surgeon, general surgeons and surgical residents. Data was collected by the principal investigators using a structured questionnaire and was analyzed by statistical package for social science (SPSS) version 23. Ethical clearance was obtained from the research review and ethical committee of the college.

## Results

A total of 2025 surgical procedures were performed in the surgical department of our institution over a period of one-year out of which colorectal and perianal procedures accounted for 14.6%. Among the total emergency and elective procedures, the share of colorectal and perianal procedures was 7.9% and 19.9% respectively. Colorectal and perianal surgeries were the second most common elective procedures next to urology surgeries in the study period. A total of 296 colorectal and perianal surgeries were performed in the study period out of which the medical records of 9 Patients could not be accessed from the medical record room making the chart retrieval rate 96.4%.

**Socio-demographic data**: Males constituted the major share of the study population in both colorectal and perianal procedure groups with 91(76.5%) and 125(80.5%) of the study subjects being male respectively. The mean age of the study population was 41.67±15.23 years with a range of 17–80 years. Patients who were operated for perianal conditions were relatively younger compared to those operated for colorectal pathologies with a mean age of 39.6±13.7 years and 44.8± 16.2 years respectively. Regarding the place of residence, only 41(17%) patients were from rural areas of the country.

**Pattern of colorectal and perianal surgeries**: Elective procedures took the major share of colorectal and perianal surgeries performed in the study period accounting for 75(63%) and 123(77.4%) of the procedures respectively. Among the colorectal disorders, redundant sigmoid was the most common indication for admission 41(34.4%) followed by colorectal neoplasms 38(31.9%) while among the perianal conditions, fistula in ano was the most common accounting for 69(43.4%) of admissions followed by hemorrhoids 35(22%) (Table 1).

**Table 1:** Diagnosis at admission, Menelik II Referral Hospital, 2018

Group of disorder	pathology	Number	Share among Admissions		Mean age	Male: female ratio
			Elective (%)	Emergency (%)		
	Redundant sigmoid	41	16(8.1%)	25(31.3%)	44.7±16.1	4.1:1
	Colorectal cancer	38	27(13.6%)	11(13.7%)	47.4±16	1.7:1
Colon and	Colostomy for reversal	18	18(9.1%)	-	41.8±15.9	17:1
rectum	Rectal prolapse	7	7(3.5%)	-	39±19.3	1.3:1
	Rectal polyp	5	5(2.5%)	-	49.8±18	4:1
	Intussusception	4	-	4(5%)	35.3±19.7	3:1
	Colorectal trauma	4	-	4(5%)	21.3±4.1	3:1
	Diverticular disease	2	2(1.1%)	-	51.6±7.2	2:1
	others	2	1(0.5%)	1(1.3%)	44±12.7	3:1
	Fistula in ano	69	69(34.8%)	-	40.8±13.7	7.6:1
	Hemorrhoids	35	35(17.7%)	-	37.2±13.3	2.5:1
Peri anal	Perianal abscess	32	-	32(40%)	37.1±12.6	4.3:1
	Anal fissure	11	11(5.5%)	-	30.1±6.9	1.2:1
	Anal stenosis	4	2(1%)	2(2.5%)	40.0±9.0	4:1
	Anal cancer	4	3(1.5%)	1(1.3%)	58.5±5.2	1:1
	Anal stricture	2	2(1.1%)	-	64±16.9	1:1

Total		278*	100%	100%		

* There were 9 patients with two pathologies that were counted separately

Overall, fistulotomy was the most common procedure accounting for 57(24.1%) followed by hemorrhoidectomy 35(12.6%) and incision and drainage for anorectal abscess 32(11.9%) Among the colorectal procedures, colostomy was the most common procedure accounting for 31(10.8%) of the total procedures (Table 2).

**Table 2:** Type of colorectal and perianal procedures, Menelik II Referral Hospital, 2018

Procedure	Frequency	Percent
Fistulotomy	57	19.9
Hemorrhoidectomy	35	12.2
Incision and drainage	32	11.1
Colostomy	31	10.8
Sigmoid colectomy and anastomosis	27	9.4
Colostomy reversal	17	5.9
Low anterior resection	12	4.2
Detorsion of sigmoid volvulus	11	3.8
Lateral sphincterotomy	9	3.1
Lt. hemicolectomy	8	2.8
Abdomino-perineal resection	7	2.4
Seton application	5	1.8
Seton exit/removal	5	1.8
Rectopexy	5	1.8
Polypectomy	5	1.8
Evaluation under anesthesia	5	1.8
Anal dilatation	4	1.4
Anoplasty	4	1.4
Rectal biopsy	4	1.4
Rt. hemicolectomy	3	1.0
Debridement	1	0.3

Total	287	100

The pre-operative diagnosis was the same with the intra operative finding in 265 (95.7%) of the procedures. Majority of the procedures were done by residents, 106 (38.1%) followed by colorectal/GI surgeon 94(33.9%). Residents handled more emergency procedures than elective procedures (80% Vs 21%).

**Figure 1:**
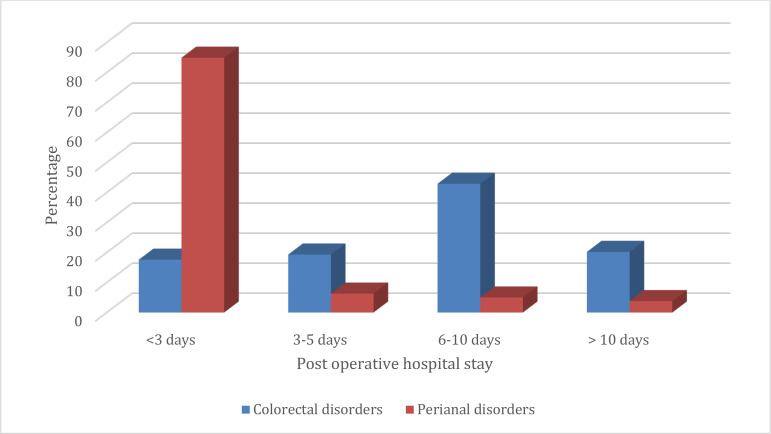
Post-operative stay after colorectal and perianal procedures

**Outcome of colorectal and perianal surgeries:** The post-operative hospital stay in the colorectal surgery group was between 6–10 days in 51(42.9%) patients whereas in the perianal surgery group majority 135(84.9%) of the patients had a post-operative stay of less than 3 days (Figure 1).

The overall incidence of post-operative complications in the colorectal and perianal procedure groups was found to be 29(24.4%) and 4(2.5%) respectively. There were 68 procedures, all in the colorectal disorders group, in which bowel anastomosis was done. Out of these, 4 developed anastomotic leak making the leak rate 5.9%. Among these 68 anastomoses, 8 were done during emergency procedures with 1 subsequent anastomotic leak and the rest 60 during elective procedures with 3 leaks making the leak rate of emergency and elective anastomosis 12.5% and 5% respectively.

Over all, surgical site infection (4.3%) was the most common post-operative complication. The incidence of surgical site infection among the colorectal procedures was 9(7.6%) while it was 3(1.9%) in the perianal procedure group. Among the study population, 7.7% underwent relaparotomy with the most common indication being anastomotic leak (35%).

There was no mortality in the perianal surgery group whereas there were 11(9.2%) deaths in the colorectal procedure group. Among these deaths, 5 occurred following elective procedures making the mortality rate 2.5% while the rest 6 were after emergency procedures with a mortality rate of 7.5%. Sepsis with multiorgan failure was responsible for 9(7.6%) of the deaths while bleeding 1(0.8) and respiratory failure 1(0.8%) were the other causes of death.

## Discussion

Colorectal and perianal surgery commands a considerable proportion of surgeon's attention in clinical practice as surgical disorders affecting the colon, rectum and the anus are quite frequently encountered (10,11). This was also true in our institution in which colorectal and perianal surgeries accounted for a fair share of the procedures done during the study period.

Benign perianal conditions (Fistula in ano, Perianal abscess, Perianal fissure, Haemorrhoid, Anal stenosis) collectively accounted for more than half of the colorectal and perianal disorders in this study. Although there are no populationbased reports regarding the prevalence of these disorders in a national or international literature, they were mentioned as growing health problems in the tropics (13,14). In one review from St. Paul's Hospital Millenium Medical College, perianal conditions were responsible for 6.4% elective surgical admissions (9).

The mean age of patients with perianal condition in our study was lower compared to the study from St. Paul's Hospital Millenium Medical college and Nepal (9,15). The most common perianal condition in this study was fistula in ano unlike studies from Nepal, India, Romania and Iran in which haemorrhoids was the leading pathology (15–18). The male predominance that was seen in our study in patients with fistula in ano as well as haemorrhoids was in line with studies from India, Egypt, Turkey, United Kingdom and Gonder (16,19–22).

In our study the most common colorectal pathology was redundant sigmoid. This is expected as this pathology has been described to be common in areas where high dietary fiber is consumed and Ethiopia and generally Africa are considered to be endemic areas (23–25). The patients with redundant sigmoid in our study were found to be younger compared to two local studies and reviews from Uganda and Turkey (9,26–28). In similar reviews on pattern of colorectal surgery admissions from the western countries, the most common pathology was colorectal malignancy. This is expected since the population-based incidence of colorectal cancer was shown to be higher in these areas compared to our country in several reports (29–31).

Colorectal surgeries are associated with higher rate of postoperative morbidity compared to other surgical subspecialities with up to one-third of patients developing these complications (32). In our study, a quarter of the patients developed complications which was lower compared to a report from Australia and France (12, 33). This difference can be explained by the retrospective nature of our study which can result in underreporting of complications which were not documented on the charts while the other two reports were prospective in design. The complication rate for perianal conditions was found to be ten times less compared to colorectal procedures in this study.

In this study anastomotic leak occurred in 5.4% of the colorectal procedures which involved bowel anastomosis. This figure compares well to another study from Ethiopia (5.2%) but higher when compared to other reports from USA (3.8%) and France (4.4%) (33–35). None of the perianal procedures required bowel anastomosis in our series.

Surgical site infection was documented in 7.6% of patients who underwent colorectal procedures in this study which was comparable to a study from Australia (7.2%) and USA (8.4%) while it was lower when compared to an earlier report from USA (11.3%) (12,2,37). Our surgical site infection rate for emergency procedures was found to be much lower when compared to a report from Japan 32.1% (38). The rate of surgical site infection for perianal procedures in our series was also found to be lower than a report from Turkey (20). These lower figures in our study may be due to underreporting of this complication. Most surgical site infections are superficial and treated conservatively in the wards and may have not been documented in the charts.

Relaparotomy was required in 7.7% of patients after colorectal procedures for various indications. This figure was lower when compared to a study from Netherland (12.5%) while it was higher than reports from Australia (5.7%) and France (2%) (36,12,33). Anastomotic leak was the most common indication for relaparotomy in our study which was also true in a similar report from Netherlands (36).

There was no mortality in patients after perianal procedures in this study which was also true in other reports from India and Turkey (16,20). The mortality rate in the colorectal surgery group was 9.2% which was higher compared to studies from France (3.4%) and USA (0.7%) (33,37). This difference in mortality can be attributed to the better health care facility including better critical care and perioperative management of patients in the western countries compared to our setup.

This study has tried to provide baseline information regarding the pattern and outcome of colorectal and perianal surgeries in the setup of a referral hospital in the capital city. We believe that this study can serve as a stepping stone for further population based prospective studies in the future to define the status of colorectal and perianal pathologies in Ethiopia better.

## Figures and Tables

**Table 3:** Post-operative complications, Menelik II Referral Hospital, 2018

Complications	Colorectal procedures				Perianal procedures				Total	
	Emergency		Elective		emergency		Elective			
	N	%	N	%	N	%	N	%	N	%
Anastomotic leak*	1	12.5	3	5	0	-	0	-	4	5.9
Surgical site infection	2	4.5	7	9.3	2	5.6	1	0.81	12	4.3
sepsis	3	6.8	1	1.3	0	-	0	-	4	1.44
bleeding	1	2.3	1	1.3	0	-	0	-	2	0.72
Sepsis+ surgical site infection+ anastomotic leak	0	-	1	1.3	0	-	0	-	1	0.36
surgical site infection+ anastomotic leak	2	4.5	0	-	0	-	0	-	2	0.72
Wound dehiscence	0	-	1	1.3	0	-	0	-	1	0.36
Stoma necrosis + Wound dehiscence	1	2.3	1	1.3	0	-	0	-	2	0.72
Stoma necrosis	2	4.5	0	-	0	-	0	-	2	0.72
Respiratory failure	1	2.3	0	-	0	-	0	-	1	0.36
Necrotizing perineal infection	0	-	0	-	1	2.8	0	-	1	0.36
Ureteric injury	1	2.3	0	-	0	-	0	-	1	0.36
Death	6	13.6	5	6.7	0	-	0	-	11	3.9

* Percentage calculated from 68 anastomoses done: 8 on emergency and 60 elective bases
